# Better under stress: Improving bacterial cellulose production by *Komagataeibacter xylinus* K2G30 (UMCC 2756) using adaptive laboratory evolution

**DOI:** 10.3389/fmicb.2022.994097

**Published:** 2022-10-12

**Authors:** Kavitha Anguluri, Salvatore La China, Marcello Brugnoli, Stefano Cassanelli, Maria Gullo

**Affiliations:** Department of Life Sciences, University of Modena and Reggio Emilia, Reggio Emilia, Italy

**Keywords:** bacterial cellulose, *Komagataeibacter xylinus*, adaptive laboratory evolution, phenotypic improvement, alternative carbon sources

## Abstract

Among naturally produced polymers, bacterial cellulose is receiving enormous attention due to remarkable properties, making it suitable for a wide range of industrial applications. However, the low yield, the instability of microbial strains and the limited knowledge of the mechanisms regulating the metabolism of producer strains, limit the large-scale production of bacterial cellulose. In this study, *Komagataeibacter xylinus* K2G30 was adapted in mannitol based medium, a carbon source that is also available in agri-food wastes. *K. xylinus* K2G30 was continuously cultured by replacing glucose with mannitol (2% w/v) for 210 days. After a starting lag-phase, in which no changes were observed in the utilization of mannitol and in bacterial cellulose production (cycles 1–25), a constant improvement of the phenotypic performances was observed from cycle 26 to cycle 30, accompanied by an increase in mannitol consumption. At cycle 30, the end-point of the experiment, bacterial cellulose yield increased by 38% in comparision compared to cycle 1. Furthermore, considering the mannitol metabolic pathway, D-fructose is an intermediate in the bioconversion of mannitol to glucose. Based on this consideration, *K. xylinus* K2G30 was tested in fructose-based medium, obtaining the same trend of bacterial cellulose production observed in mannitol medium. The adaptive laboratory evolution approach used in this study was suitable for the phenotypic improvement of *K. xylinus* K2G30 in bacterial cellulose production. Metabolic versatility of the strain was confirmed by the increase in bacterial cellulose production from D-fructose-based medium. Moreover, the adaptation on mannitol did not occur at the expense of glucose, confirming the versatility of K2G30 in producing bacterial cellulose from different carbon sources. Results of this study contribute to the knowledge for designing new strategies, as an alternative to the genetic engineering approach, for bacterial cellulose production.

## Introduction

Naturally produced polymers are characterized by impressive traits. They are produced by living organisms, converting suitable raw materials into large and complex polymeric structures. Biopolymers are produced both by the biggest living organisms, such as plants, and by the smallest living cells, as bacteria (Kaplan, 1998). Referring to the bacterial life cycle, one of the main mechanisms adopted by bacteria to resist to environmental stressors is by biofilm formation, a biomass consisting of microbial cells immersed in an exopolysaccharidic matrix (Moradali and Rehm, 2020). Among biofilm exopolysaccharides, bacterial cellulose (BC) was massively studied in the last decades, due to its natural properties of interest for many industrial applications (La China et al., 2021; Manan et al., 2022). Unlike plant cellulose, BC is an ultrapure biopolymer based on glucose monomers linked by β-1,4 glycosidic bond. BC is synthetized by a membrane protein complex, named cellulose synthase (CS), constituted by four main subunits (BcsA, BcsB, BcsC, BcsD). The glucan chain is synthetized by the catalytic core of CS (BcsA and BcsB), in which activated glucose monomers (UDP-glucose) are linked to the native glucan chain (Gullo et al., 2018). The glucan chain is extruded *via* structural proteins of CS (BcsC and BcsD), which form a channel in the periplasmic space and outer membrane. The extrusion point of the native glucan chain in the environmental space is called terminal complex and it is a distinct site on the surface of the outer membrane (Manan et al., 2022). The final structure of BC is the result of assembling events occurring in the extracellular space of cells. Briefly, the first assembly event occur between glucan chains forming protofibrils of about 2–20 nm in diameter. Subsequent events involve the protofibril assembly, by forming the ribbon shaped microfibrils *via* the formation of hydrogen bonds and Van der Waals forces, and finally the 3D hierarchical network of bundles (Gullo et al., 2018; Kim et al., 2019). The hierarchical polymerization of the glucan chains confers to BC unique properties, such as high crystallinity and high tensile strength (Singhania et al., 2021). Such properties make BC suitable for a wide range of applications, spanning from food (Vigentini et al., 2019), biomedical (Brugnoli et al., 2021; Manan et al., 2022) and cosmetics fields (Ullah et al., 2015). The most performant BC producers are found within the *Acetobacteraceae* family. This bacterial family includes species that are able to oxidize carbon substrates resulting in the production of a wide range of carboxylic acids (La China et al., 2018; Stasiak-Różańska and Płoska, 2018). Industrial processes in which *Acetobacteraceae* members of the acetic acid bacteria (AAB) group are exploited, include fermented beverages production, where BC is an undesired compound (Giudici et al., 2009; Gullo et al., 2016). Within AAB, strains belonging to the *Komagataeibacter* genus are not only interesting for fermentation processes because of their high aptitude in producing organic acids (Wang et al., 2015), but are widely studied for their ability in producing BC (Gullo et al., 2018; La China et al., 2021; Vadanan et al., 2022). At least six species of *Komagataeibacter* were described as BC producers, namely *K. europaeus, K. hansenii, K. rhaeticus, K. medellinensis, K. melomenusus,* and *K. xylinus*. The above-mentioned species were described to achieve variable and considerable BC yield and be able to metabolize different carbon sources for BC production (Masaoka et al., 1993; Keshk and Sameshima, 2005). Among the described species of *Komagataeibacter,* high intra and inter-species variability in term of BC yield was observed (Brugnoli et al., 2021; La China et al., 2021). However, *K. xylinus* species is considered as the model organism for BC production and the highest producer in different conditions (Wang et al., 2018; Zhong, 2020). Indeed, many efforts for scaling-up BC production are directed to the strain selection, especially considering the intra-species variability within *K. xylinus* (Gullo et al., 2017; Sharma and Bhardwaj, 2019; Andriani et al., 2020; Ryngajłło et al., 2020). Different approaches were assayed, mainly by replacing the carbon source with alternative substrates, also derived from wastes, such as agro-food, contributing to the development of more sustainable bioprocesses (Islam et al., 2017, 2020; Machado et al., 2018; Wang et al., 2018; Raiszadeh-Jahromi et al., 2020; Li et al., 2021a). Using cell-free system, an improved yield was observed compared to BC produced by microbial cells, consuming less carbon source and, consequently, reducing production costs (Ullah et al., 2015). Other approaches were assessed by developing synthetic biology tools, modifying metabolic pathways involved in BC synthesis (Ryngajłło et al., 2020; Singh et al., 2020). An alternative approach to genetic engineering strategy is the exploitation of adaptation mechanisms, developed by bacteria under predefined environmental conditions (Lee and Palsson, 2010; Barve and Wagner, 2013; Matsutani et al., 2013; Zorraquino et al., 2016). The activation of programs that regulate the cell physiology to the new environmental condition, is provided by metabolite concentration, energy levels or physical stressors, tuning the gene expression with the physiological needs (López-Maury et al., 2008). The modulation plasticity of the adaptation programs is depending both on the environmental changes and on the bacterial species (Hindré et al., 2012).

Here we have assayed the adaptation ability of *K. xylinus* K2G30 to mannitol as an alternative carbon source by applying the adaptive laboratory evolution (ALE) approach. Glucose, conventionally known as the main building block for BC production, was replaced by mannitol, already tested in *K. xylinus* K2G30, achieving an improvement of BC yield (Gullo et al., 2019). In addition, the choice of mannitol as carbon source to test the adaptation abilities in *K. xylinus* lies in the metabolic pathway, by which mannitol is converted into glucose for BC production. *K. xylinus* K2G30 was repeatedly cultivated using mannitol as sole carbon source along 210 days. The carbon sources consumption and the production of organic acids were evaluated. Structural changes over adaptation cycles, including X-ray diffraction (XRD), network frame variation, crystallinity, and fiber size, were evaluated by using scanning electron microscopy (SEM).

## Materials and methods

### Strain and culture conditions

**Figure 1:**
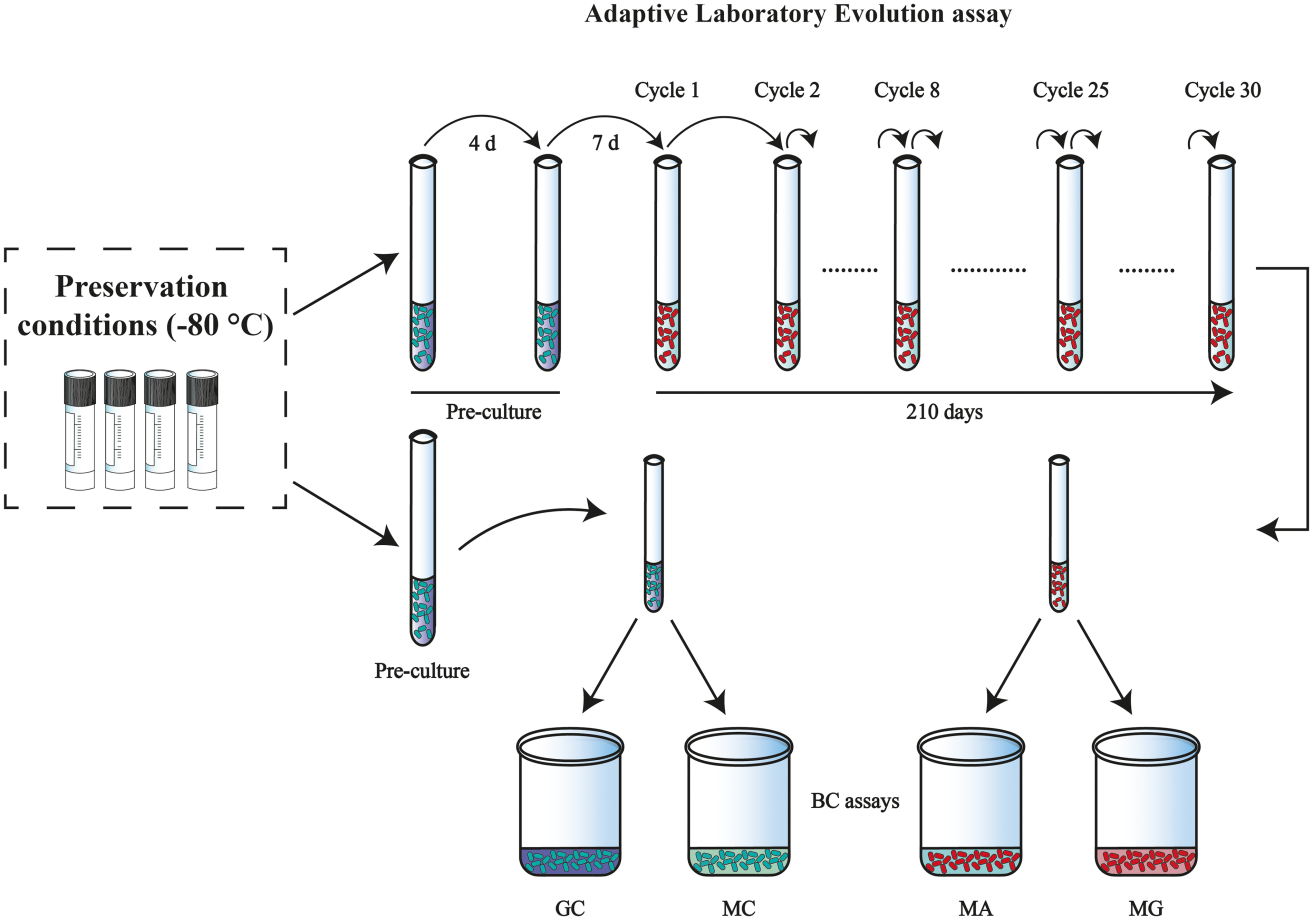
Overview of the experimental design: adaptation induction of *Komagataeibacter xylinus* K2G30 in mannitol medium. Preculture was maintained in HS-G and adaptation of the strain in mannitol medium begins from cycle 1. Both the adapted and the parental strain were tested for BC quantification and each color represents a different condition: MA (adapted in mannitol medium), MG (adapted strain in glucose medium), GC (parental strain in glucose medium), and MC (parental strain in mannitol medium).

The parental strain *K. xylinus* K2G30 used in this study had previously been isolated from kombucha tea (Mamlouk, 2012). The strain was deposited in UMCC (Unimore Microbial Culture Collection) under the collection code UMCC 2756 (Gullo et al., 2019). All the experiments were carried out in Hestrin–Schramm (HS) medium, which consists of yeast extract 1% w/v, polypeptone 0.5% w/v, disodium phosphate anhydrous 0.27% w/v, citric acid monohydrate 0.115% w/v, supplemented with D-glucose (standard) or D-mannitol (alternative), at the final concentration of 2% w/v (Hestrin and Schramm, 1954). A subculture of K2G30 was generated by rehydrating a glycerol (50% v/v) stock from −80°C preservation condition. From this subculture a total of 60 new glycerol stocks were prepared as control for the experiment of adaption cycles (Figure 1).

### Adaptive laboratory evolution

ALE was carried out as per the experimental design referring to Figure 1. To establish the evolutionary experiment, the parental *K. xylinus* K2G30 strain was revitalized in 5 ml of HS-Glucose (HS-G) medium and cultivated for 4 days at 28°C, under static conditions. Preculture was prepared by inoculating the refreshed culture into 10 ml of fresh HS-G medium, the incubation was performed at 28°C for 7 days. This preculture was used to initiate the adaptation in mannitol, where the spent medium was removed by centrifugation at 8000 g for 5 min at 4°C. Cells were washed twice and refreshed with the HS-mannitol (HS-M) medium, and this inoculum was assayed at OD_600_. With the known absorbance value (0.01), 0.5 ml (5% v/v) of the inoculum (HS-M) was inoculated into three test tubes, each containing 10 ml of fresh HS-M medium and incubated at 28°C for 7 days. Continuous adaptation of the strain to the new carbon source was performed by transferring the culture to a fresh medium every 7 days, washing the cells as described before, and transferring 0.5 ml (5% v/v) of the inoculum. The refreshing step is referred to as “cycles of adaptation,” in this study. Adaptation was carried out up to 30 cycles, with 1 to 8 cycles considered for short-term adaptation, and 25 and 30 cycles were tested for long-term adaptation. Control conditions were maintained constant for every cycle and performed using subcultures generated, as previously described.

### Bacterial cellulose production

BC production assay was performed in four individual conditions as illustrated in Figure 1, by removing the spent culture medium by centrifugation at 8000 g for 5 min. Pellets were washed twice, refreshed in the representative medium (HS-G, HS-M) and OD_600_ was taken before the culture strains were inoculated. A total of 3 ml of these cultures was inoculated in 60 ml of the carbon source (glucose or mannitol) medium and incubated at 28°C for 5 days, under static conditions. All the experiments were performed with three biological replicates.

### Bacterial cellulose harvesting, purification, and quantification

Native BC pellicles were separated from the culture broth, washed with distilled water to remove the medium components, and treated with 1 M NaOH, for 30 min at 80°C. Further, the pellicles were washed with distilled water until neutral pH and dried at 20°C with air flow, until the constant weight of cellulose layer was reached. Dried BC layers were weighed and analyzed as described by Gullo et al., 2017. The residual culture medium was collected immediately and stored at −20°C for further analysis.

### Evaluation of carbon source consumption and gluconic acid production over the adaptation cycles

Residual concentration of mannitol and glucose in the culture medium were quantified by using K-MANOL and K-SUCGL assay kits (Megazyme Ltd. Bray, Ireland), respectively. Furthermore, gluconic acid was determined by the enzymatic kit from K-GATE, Megazyme Ltd. Bray, Ireland. Analyses were performed according to the manufacturer instructions and calculated using MEGA-CALC. The pH of the medium was determined by using an automatic titrator (TitroLine EASY SCHOTT Instruments GmbH. Mainz, Germany). All the data were expressed as gram per liter (mean ± standard deviation).

### Bacterial cellulose production using D-fructose as carbon source in the adapted strain

*K. xylinus* K2G30 strain adapted in mannitol for 30 cycles was tested in HS medium supplemented with D-fructose (HS-F) instead of D-Mannitol and compared with the parental strain. Quantification of BC was performed as described above. Residual D-Fructose was quantified by using the Megazyme kit (K-SUFRG, Megazyme Ltd. Bray, Ireland) according to manufacturer instructions and calculated using MEGA-CALC. Genes and proteins sequences were obtained by downloading the already sequenced *K. xylinus* K2G30 genome from NCBI database (assembly id GCA_004302915.1) and performed the structural and functional annotation using Prodigal v2.6.3 (Hyatt et al., 2010) and Prokka v1.13.4 (Seemann, 2014), respectively. The pathway reconstruction was performed using KEGG database.

### Structural characterization of bacterial cellulose layers

The surface morphology of dried BC layers were observed by SEM (NovaNano SEM 450, FEI, United States), as described by Mohammadkazemi et al. (2015) with some modifications. Briefly, SEM was performed in high vacuum mode with an acceleration voltage of 10 kV. All the samples were coated with a layer of gold to improve conductivity. Fiber dimension was estimated by randomly measuring the diameter of 100 fibers using ImageJ software (ImageJ 1.53 k version) (Schneider et al., 2012).

Dried BC layers were X-rayed using a film diffractometer (X’Pert PRO, Marvel Panalytical, United Kingdom). XRD patterns of the samples were recorded using CuKα radiation (λ = 1.54 Å), at a voltage of 40 kV and a filament emission of 40 mA. Samples were scanned with ramping at 1° min^−1^, analyzing the range of 10°–30° (2θ). A zero-background holder was used to avoid the detection of any peak not related/associated to the sample. The crystallinity index was calculated using the following formula:


Equation 1 $$cr\left(\%\right)=\frac{sc}{st}*100$$

Where *s_c_* is the sum of net area and s_t_ is the sum of total area (Mohammadkazemi et al., 2015).

## Results and discussion

### Adaptive laboratory evolution to increase the bacterial cellulose yield in *Komagataeibacter xylinus* K2G30

To optimize BC production, different approaches have been implemented in the past, by using alternative carbon sources, also deriving from wastes, with the aim of reducing the production cost (Jozala et al., 2015; Kadier et al., 2021). In this context, it is possible to depict main features that a rapresentative carbon source should possess: fits the metabolic needs of the bacterial strain; guarantees high yield; be available in wastes.

Mannitol was previously tested in *Komagataeibacter* strains, obtaining an increasing of BC yield (Oikawa et al., 1995; Mohammadkazemi et al., 2015). In our previous studies on *K. xylinus* K2G30 and K1G4 strains, we observed an increase of BC production of about 42 and 21%, respectively, compared to glucose, highlighting the suitability of mannitol for BC production (Gullo et al., 2019; La China et al., 2020). Furthermore, *K. xylinus* K2G30 was previously tested in mannitol-based medium under shaking conditions. Cultivation conditions were different respect to that of the present study; however, BC was formed as spheres and a low yield was obtained (Gullo et al., 2019). This evidence is confirmed by previous studies aimed at evaluating the effect of dissolved oxygen on BC yield in agitated culture. The decrease of BC production in agitated conditions was correlated to the genetic instability of strains and the overgrowth of non-cellulose producers. Moreover, the increase of viscosity, proportional to BC production in agitated system, seems responsible for the non-homogeneous air bubbles dispersion, affecting the oxygen availability (Hwang et al., 1999).

Based on these considerations, mannitol was chosen as selective carbon source to adapt *K. xylinus* K2G30 by using ALE approach, in static regime. The strain was propagated in mannitol medium (condition MA) for 30 cycles (210 days). The evaluation of the strain adaptability to the new condition was assayed by measuring the BC produced during each cycle of adaptation. To monitor the ALE condition and the efficacy of the experimental design, two control conditions were used, by the cultivation of parental strain in standard conditions, using glucose as primary carbon source (GC condition), and mannitol medium (MC condition), both for every cycle (Figure 1). To evaluate if the parental phenotype persisted, the adapted strain was cultured in standard medium, using glucose as carbon source (MG condition).

**Figure 2:**
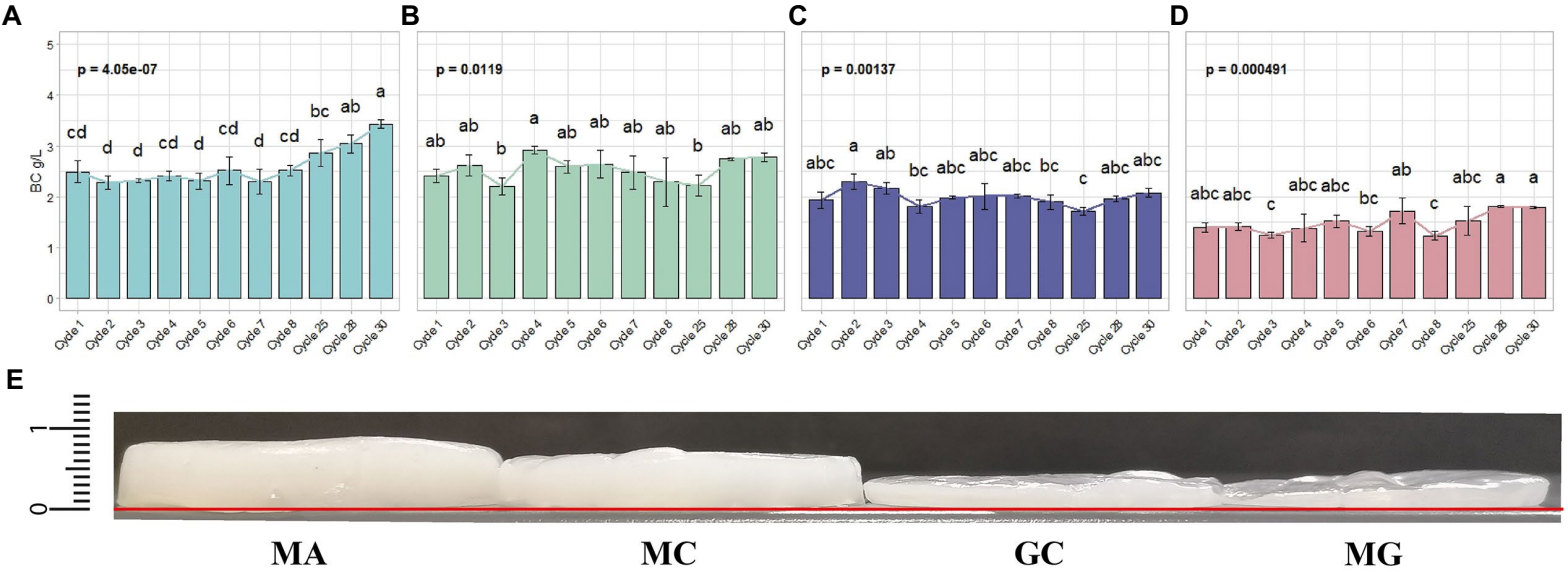
Quantification of BC yield in the four conditions tested **(A)** MA **(B)** MC **(C)** GC **(D)** MG. Each bar indicating the mean ± SD of BC yield in the representative cycles. Significant difference among the BC yield was represented by letters. **(E)** Layers thickness of native BC pellicles at cycle 30: MA = 0.85 mm; MC = 0.70; GC = 0.40; MG = 0.40. The baseline was marked in red.

In mannitol (MA condition), the amount of BC produced during the first adaptation cycles (cycles from 1 to 8) remained unchaged (Figure 2A). This condition persisted until cycle 25. Starting from this cycle, we observed a constant increase of produced BC. At cycle 28, BC yield was 3.04 ± 0.17 g/l, reaching 3.43 ± 0.09 g/l of BC at cycle 30. Regarding glucose control (GC condition) no significant changes were observed over the cycles (Figure 2B). The BC produced in this condition ranged between 1.71 ± 0.08 g/l (at cycle 25) to 2.30 ± 0.15 g/l (at cycle 2), corresponding to the minimum and maximum among all the tested cycles, respectively. Also in the case of mannitol control (MC) no significant differences in BC production were observed over the cycle (Figure 2C). We tested also the condition in which *K. xylinus* K2G30 in MA condition, was cultured back in glucose medium (MG condition). The BC yield obtained over the cycles does not show significant differences (Figure 2D). Furthermore, in MG condition, a low yield of BC production was observed, compared to glucose control (GC condition), producing 1.45 ± 0.18 g/l on average in over all cycles, while 1.99 ± 0.17 g/l of BC were observed in GC condition. The low yield observed in MG, corresponded to a loss of 27.13% of BC compared to the parental strain. Bacterial cellulose layers obtained during the adaptation cycles were illustrated in Figure 2E, highlighting the differences described. This behaviour could be explained considering the bet-hedging strategy that bacteria adopt to optimize their fitness in the environment (Solopova et al., 2014). The bet-hedging describes the strategies adopted by individuals in a population to optimize their fitness in a specific environment (Olofsson et al., 2009). The idea behind the bet-hedging theory is that individuals tend to reduce their variance in fitness to increase their fitness in long-term (Monod, 1949; Slatkin, 1974; Olofsson et al., 2009). When bacteria are cultivated in a different condition than stardard (glucose is considered the standard carbon source for most of bacteria), their metabolism is modulated in order to increase their survival rate. If multiple carbon sources are supplied in the medium, a growth transition usually occur, characterized by the growth interruption. This step is followed by a growth recovery in which bacterial cells switch to the less “attractive” carbon source. The interruption of cell growth (known as diauxic lag-phase) represents the period in which bacteria apply changes in their enzimatic sets, shifting to the metabolic pathways related to the new carbon source (Stanier, 1951). The diauxic and bet-hedging were widely studied in *Escherichia coli* (Wang et al., 2019) and *Lactobacillus lactis* (Solopova et al., 2014). In this study, by repeated culturing *K. xylinus* K2G30 in mannitol, we can speculate that its metabolism was reprogrammed to use mannitol as main carbon source. By re-cultivating the adapted *K. xylinus* K2G30 strain in glucose, we observed a reduction of the BC yield, highlighting a metabolic shift. Previous works related to ALE approach to improve BC production in *Komagataeibacter* strains were focused on the description of genetic mutation related to the metabolic shift. *K. medellinensis* NBRC 3288 was repeatedly cultured to increase the BC production. The genomic analysis revealed mutations involving 11 proteins involved in BC biosynthesis (Matsutani et al., 2015). In a recent work, performed on *K. xylinus* MSKU12, the ALE approach was used to increase the BC yield using different modified media. In coconut water-based medium, an increase of BC starting from 140 days and which further improvement until the end of the tested period (i.e. 210 days) (Naloka et al., 2020). Our results are in accordance with the adaptation time of *K. xylinus* MSKU12, showing a slow and steady improvement of BC production using an alternative carbon source. These data highlight the time frame required for the metabolic shift in *K. xylinus* strain.

### Substrate uptake rates and production of byproduct in the adapted *Komagataeibacter xylinus* K2G30

**Figure 3:**
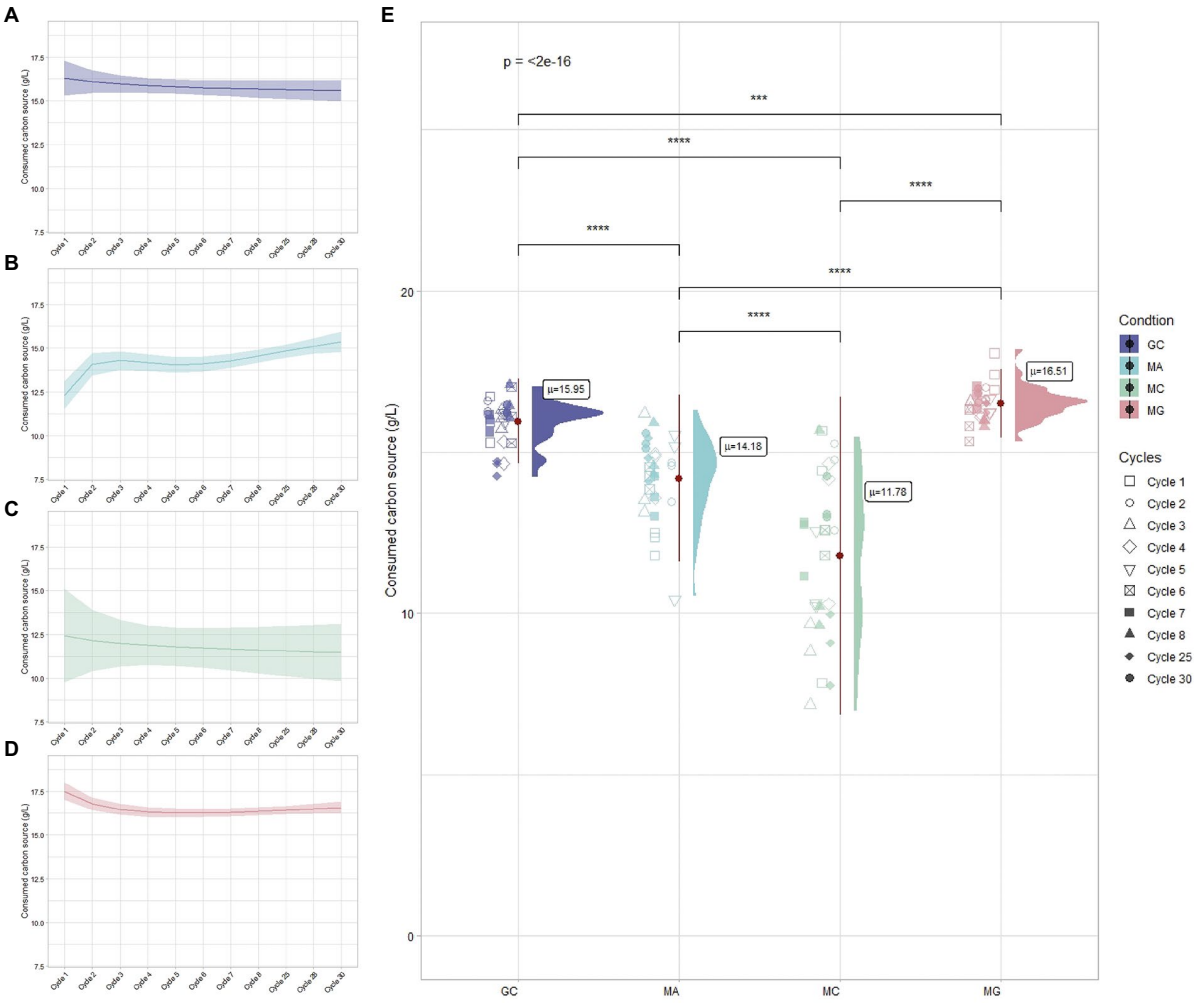
Carbon source utilization during the ALE cycles for **(A)** GC, **(B)** MA, **(C)** MC, and **(D)** MG conditions. **(E)** Overall carbon source consumption among conditions: each points represents the carbon source replicates related to the cycle and “μ” indicates the average consumption of carbon source among tested cycles.

The BC production occurs through the consumption of carbon sources supplemented in the medium. *Komagataeibacter* strains are able to use a wide range of sugars or sugar-alcohol for BC production (Mikkelsen et al., 2009; Mohammadkazemi et al., 2015; Gullo et al., 2019). The efficiency of the carbon source utilization for BC production is strictly influenced by the metabolic status of bacterial cells and the availability of dedicated metabolic pathways (Nguyen et al., 2021a). The utilization of mannitol as carbon source for BC biosynthesis varies among *Komagataeibacter* species. Ours previous work on *K. xylinus* K2G30 and K1G4 strains highlighted the highest BC production in mannitol-based medium (Gullo et al., 2019; La China et al., 2020). In contrast, a recent work on *K. uvaceti* reported a low BC synthesis in mannitol media (Nascimento et al., 2021). In this study, consumption of carbon sources during the adaptation cycles were monitored for the four conditions (MA, MG, MC, and GC) and were represented in Figure 3. A comparison between cycle 1 and cycle 30, based on BC produced and carbon source consumed, was reported in Table 1. When *K. xylinus* K2G30 was repeatedly cultivated in mannitol-based medium, the initial cycles were characterized by low consumption of the carbon source (cycle 1, 12.22 ± 0.42 g/l; Figure 3B). An increased mannitol utilization was observed between cycle 1 and cycle 2 (14.23 ± 0.68 g/l), remaining unchanged until cycle 25. A further increase of mannitol consumption has been observed in cycle 28 (15.31 ± 1.28 g/l) and cycle 30 (15.35 ± 0.18 g/l). The carbon source consumption in MA condition follows the same trend of BC production. This increase in the carbon source utilization could be explained considering the diauxic shift occurring in bacteria when they are cultivated in a multiple carbon source media. The metabolic shift is a time-consuming process, occurring during the continuous exposure to a carbon source by the modulation of the enzymatic set (Solopova et al., 2014; Bajic and Sanchez, 2020). This phenotype is highly visible considering the MC condition, in which high variability in mannitol consumption was observed (Figure 3E). Indeed, the consumption of the carbon source in the parental strain varied between 12.57 ± 3.82 g/l at cycle 1 to 13.46 ± 0.68 g/l at cycle 30, characterized by fluctuations in every cycle (Figure 3C). Such fluctuations, reflecting the BC production trend described in the previous section, could be the results of the adaptation process at early stage. A study conducted on *Lactococcus lactis* highlighted a heterogenicity in the cell population when cultivated in a multiple carbon source medium (glucose and cellobiose; Solopova et al., 2014): a cell fraction was able to utilize the favorable carbon source, while the remaining fraction utilized the new carbon source. This heterogeneity is the result of the dynamic mechanisms of the adaptation process occurring during the adaptation period, in which is possible to distinguish adaptive “driver” events (as genetic mutations) and non-adaptive events (mutation not involved in the adaptation response; Numata et al., 2019). Based on this consideration, we can speculate that regulatory mechanisms act in the metabolic shifting from glucose to mannitol, determining heterogenicity in *K. xylinus* K2G30 population.

**Table 1:** Comparison between 1 and 30 cycles in term of BC produced, consumed carbon source, gluconic acid, and pH.

SAMPLES	BC yield (g/L)		Consumed CS (g/L)		Gluconic acid		pH	
	1 cycle	30 cycle	1 cycle	30 cycle	1 cycle	30 cycle	1 cycle	30 cycle
MA	2.49 ± 0.22	3.43 ± 0.09	12.22 ± 0.42	15.35 ± 0.18	0.04 ± 0.02	0.08 ± 0.02	6.25 ± 0.01	6.37 ± 0.05
MC	2.41 ± 0.14	2.77 ± 0.08	12.57 ± 3.82	13.46 ± 0.68	0.09 ± 0.01	0.01 ± 0.05	6.32 ± 0.04	6.27 ± 0.02
GC	1.94 ± 0.17	2.08 ± 0.09	16.02 ± 0.58	16.3 ± 0.16	8.33 ± 0.65	7.65 ± 0.77	3.6 ± 0.06	3.49 ± 0.02
MG	1.4 ± 0.1	1.8 ± 0.02	17.55 ± 0.6	16.78 ± 0.19	7.43 ± 1.79	4.87 ± 0.45	3.55 ± 0.04	3.39 ± 0.01

Data were reported as mean ± standard deviation.

While the parental strain cultivated in glucose-based medium (GC condition) showed the same consumption rate of the carbon sources (15.95 ± 0.69 g/l) among cycles (Figure 3A), an increase of the consumption rate has been observed when the adapted strain was cultivated in glucose (MG condition), resulting in average consumption of 16.51 ± 0.45 g/l of glucose (Figure 3E). A reduction in glucose consumption has been observed between cycle 1 and cycle 2, remaining stable for the successive cycles (Figure 3D). The increased consumed carbon source does not reflect the BC production, for which MG resulted to be the lower BC yield condition (Table 1). The reduction of the carbon source utilization in the BC biosynthetic pathway could be explained by considering the survival strategies adopted by bacteria under stress condition. The exposure to an environmental change induces in bacteria physiological and translational events to tune the molecular machinery to the new environmental condition (Nguyen et al., 2021b). Two possible scenarios are possible based on the environmental conditions. The exposure to a fluctuating environment, for example rapid changes (in minutes timescale) in nutrient concentrations, causes the activation of extremely rapid responses to the nutrient fluctuations. In contrast, the exposure to a steady environment, as a nutrient shift, lead to a new steady growth state (Nguyen et al., 2021a). The second scenario may explain the MG condition, in which *K. xylinus* K2G30 cells, repeatedly exposed to a nutrient shift (glucose to mannitol), reached the steady growth state in mannitol-based medium but not in glucose-based medium. We can hypothesize that the amount of carbon source consumed was not used for BC production but was consumed to adjust the cell physiology to the new shift in carbon source (mannitol to glucose).

In this study, also the byproduct formation was evaluated, mainly represented by gluconic acid formation (Supplementary Table S1). Formation of organic acids is a peculiar trait of AAB during the oxidative fermentation (Gullo et al., 2018). A massive production of organic acids leads to a reduction of environmental pH, representing a stressor for the bacterial culture and affecting the BC production (Li et al., 2021b). By using mannitol (condition MA and MC), no production of gluconic acid was detected. When glucose was used as carbon source (GC and MG) a high production of gluconic acid was observed, particularly in GC condition. In this condition, *K. xylinus* K2G30 cells are exposed to stress condition, due to the acidification of the environment. Furthermore, since gluconic acid is produced starting from glucose by the membrane-bound glucose dehydrogenase EC 1.1.5.2, the availability of the carbon source resulted limited (Gullo et al., 2018). It is important to highlight the production of gluconic acid in MG condition. At cycle 1, the production was similar to GC, but focusing on cycle 30 a reduced amount of gluconic acid was produced. This could be attributed to the adaptation mechanism, valorizing our hypothesis that a metabolic shift occurred in the late stages of the adaptation. Furthermore, the reduction of gluconic acid observed at cycle 30 in MG was not accompanied by an increase of pH (Table 1).

### Bacterial cellulose production in D-fructose-based medium improved by adaptive laboratory evolution

**Figure 4:**
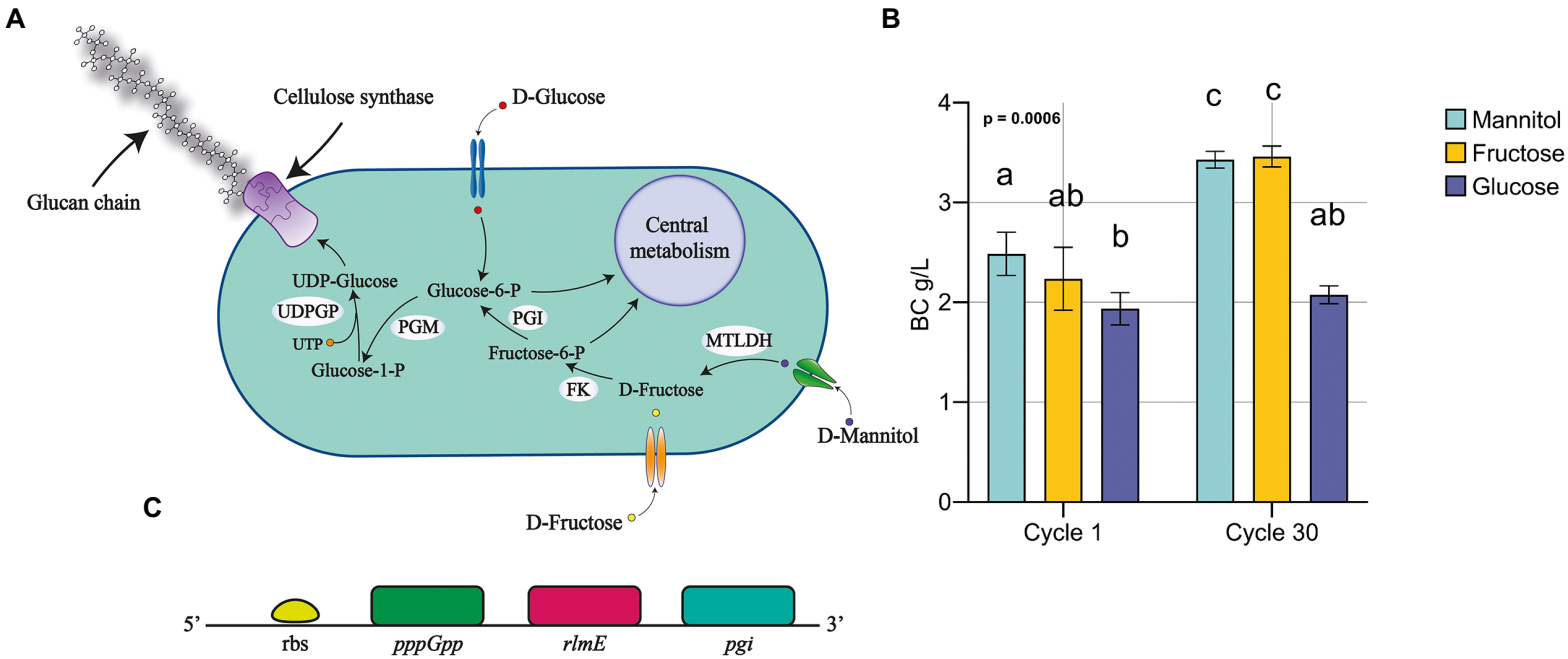
Fructose utilization as carbon source for BC production: **(A)** schematic illustration of fructose and mannose metabolic pathway, focusing on the bioconversion of mannitol for BC production; **(B)** BC production using fructose and comparison among MA, MF and GC. Data were represented as mean ± standard deviation. Tukey test was used to assess multiple comparison among conditions. Different letters indicate statistical differences among conditions (*p* < 0.05); **(C)** schematic representation of *pgi* related gene disposition in *K. xylinus* K2G30 genome.

So far, we described the phenotypic improvement of *K. xylinus* K2G30 by applying the ALE approach. At this stage we focused on mannitol pathway to deeply understand the metabolic reactions involved in the conversion of mannitol to UDP-glucose, the activated substrate utilized for BC biosynthesis. A schematic representation of the pathway is illustrated in Figure 4A. Enzymes involved in the catabolism of mannitol and production of BC are listed in Table 2.

**Table 2:** Metabolic pathway of mannitol metabolism for the biosynthesis of BC.

Enzyme	Gene	Enzyme	EC	Pathway	Gene coordinate (bp) (strand)
MTLDH	*mtlK*	Mannitol 2-dehydrogenase	1.1.1.67	Fructose and mannose metabolism	84,394–85,875 (+)
FK	*srcK*	Fructokinase	2.7.1.4	Fructose and mannose metabolism	150,700–151,629 (−)
PGI	*pgi*	Phosphoglucose isomerase	5.3.1.9	Starch and sucrose metabolism	53,428–56,292 (+) 3,784–6,642(−)
PGM	*pgm*	Phosphoglucomutase	5.4.2.2	Starch and sucrose metabolism	12,962–14,620 (−)
UDPGP	*galU*	UTP--glucose-1-phosphate uridylyltransferase	2.7.7.9	Starch and sucrose metabolism	51,546–52,415 (+)
BcsI	*bglB_2* *bcsD* *bcsC* *bcsB* *bcsA* *ccpax* *cmcax*	Cellulose synthase (operon I)	3.2.1.21 – – – 2.4.1.12 – 3.2.1.4	Starch and sucrose metabolism	256,838–259,045 (−) 259,263–259,733 (−) 259,733–263,695 (−) 263,698–266,121 (−) 266,108–268,372 (−) 268,550–269,545 (−) 269,542–270,633 (−)
BcsII	*bcsAB* *bcsX* *bcsY* *bcsC*	Cellulose synthase (operon II)	– – 2.3.1.- –	Starch and sucrose metabolism	5,579–10,150 (+) 10,265–10,936 (+) 11,023–12,183 (+) 12,266–16,126 (+)
BcsIII	*bcsAB* *bcsC*	Cellulose synthase (operon III)	– –	Starch and sucrose metabolism	28,895–33,346 (+) 33,349–37,149 (+)
bcsAB	*bcsAB*	Cellulose synthase	–	Starch and sucrose metabolism	83,990–88,687 (+)

The enzymatic set was retrieved analyzing the *K. xylinus* K2G30 previously reported (Gullo et al., 2019). The table reports the enzymes and relative genes involved in the bioconversion of mannitol until the formation of the activated glucose monomer (UDP-Glucose). The number of BC operons described in the *K. xylinus* K2G30 genome were reported as well. Furthermore, the gene coordinates were specified.

Based on the pathway reconstruction on the previous analysis on *K. xylinus* K2G30 genome and the reconstruction using KEGG, mannitol is the substrate of mannitol-2-dehydrogenase (MTLDH), converting the D-mannitol in to D-fructose. It will be converted in fructose-6-phosphate by the activity of a fructokinase (FK) and in glucose-6-phosphate by the phosphoglucose isomerase (PGI). The reaction that provides glucose monomers to BC biosynthesis is the isomerization of the glucose-6-phosphate to glucose-1-phosphate by the phosphoglucomutase (PGM). This pathway was also described previously (Zikmanis et al., 2021).

Based on this description, it is reasonable to hypothesize that if *K. xylinus* K2G30 was adapted in mannitol, possibly, the adaptation effect should be visible also supplementing fructose as carbon source. We tested the *K. xylinus* K2G30 from cycle 30 in MA condition in D-fructose based medium (MF condition) and the parental strain, comparing the BC yield with MA and GC at cycle 1 and cycle 30 (Figure 4B). As expected, at cycle 1 the only appreciable difference was related to the comparison MA versus GC. Considering cycle 30, MF showed the same improvement described for MA in the previous section, producing 3.46 ± 0.1 g/l (MF) and 3.43 ± 0.09 g/l (MA). No changes in gluconic acid production and pH were observed in MF and MA conditions (Supplementary Table S2). The observed increase of production in MF condition could reflect an up-regulation of the fructose-mannose metabolic pathway caused by the continuous cultivation in mannitol. By looking at the structural annotation of *K. xylinus K2G30 genome*, the *pgi* gene are deprived of a ribosomal binding site (rbs) (gene coordinates 150,700–151,629), suggesting that the regulation of the transcription mechanism of this gene is depending on the rbs of another genes. The first available rbs has been found two genes upstream and is related to the gene coding for the guanosine-5′-triphosphate 3′-diphosphate pyrophosphatase (pppGpp) (Figure 4C). This enzyme was identified as one of the key enzymes in the modulation of to growing-limiting conditions adopted by bacteria (Steinchen et al., 2020). High level of pppGpp induce a process recognized as “stringent response,” a major cellular reprogramming in which the rRNA and tRNA synthesis is down regulated, stress-related genes upregulated and the limiting resource allocation is optimized (Atkinson et al., 2011; Gaca et al., 2013). Depending on the pppGpp rbs, an upregulation of the *pgi* gene is possible, increasing the conversion rate of fructose-6-phosphate in to glucose-6-phosphate from fructose-6-phosphate. When microbial cells are transferred into an environment with harmful pressure, they evolve the target metabolic pathway to enhance the growth condition, by exploiting the most abundant resources (Tan et al., 2019). As an example, in *E. coli* an increase in the β-lactamase metabolic pathway by continuous exposure to β-lactam was obtained (Abraham and Chain, 1940; Fevre et al., 2005). The up-regulation of target metabolic pathways as adaptation mechanisms were also reported in yeast (Hong et al., 2011). Nevertheless, in bacteria, the adaptation mechanisms when they are exposed to specific environmental condition have been described highly complex, in which genetic mutations, up-regulation processes, and metabolic arrangements are finely tuned (Connolly et al., 2021).

### Structural and optical changes in bacterial cellulose layers during the adaptation cycles

**Figure 5:**
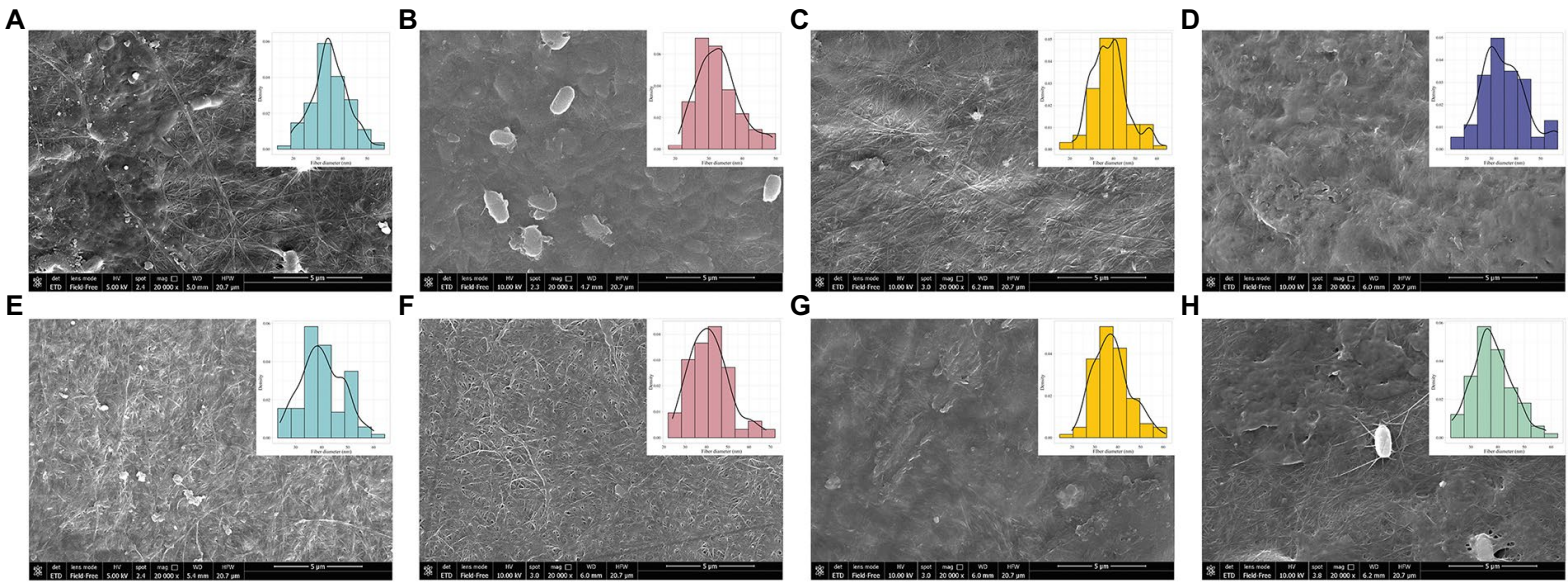
BC morphology characterization among conditions by using SEM. The characterization was performed considering cycle 1 and cycle 30 for **(A–E)** MA, **(B–F)** MG and **(C–G)** MF conditions. Only sample from the cycle 1 were assayed for **(D)** GC and **(H)** MC conditions.

Mechanical properties of the BC are mostly dependent on the ultrafine structure and arrangement of the nanofibers (Mohammadkazemi et al., 2015). BC structure rearrangements, especially in fiber diameter, were reported in previous work as a consequence of changes in the culture conditions (Li et al., 2021a). For this reason, we evaluated the morphology of BC layers during the adaptation cycles using SEM. A set of comparisons between cycle 1 and 30 for the conditions MA (Figures 5A–E), MG (Figures 5B–F) and MF (Figures 5C–G) was conducted. Fibrous networks were compared with GC (Figure 5D) and MC (Figure 5H). From Figure 5, it is possible to observe the presence of bacterial cells entrapped in the cellulose layer (Figures 5A,B,H). This is an eventuality that can occur as previously reported by Hult et al. (2003) and Yim et al. (2017), which observed that the treatment with 1 M NaOH solution guarantees the death/disruption of bacterial cells but in some cases, not the total removal. In this study, SEM imaging revealed a heterogenous rod shaped nanofibrillar structure in all the samples and, in some cases, noticeable fibers agglomerates. However, a more compact fiber arrangement could be attributable to fibers random assembling (Sheykhnazari et al., 2011; Mohammadkazemi et al., 2015) or even to a failed integration of the microfibrils to the nascent BC sheet (Nicolas et al., 2011). At cycle 1, comparing the diameter of the fibers among the conditions, no differences were observed among them by using the three carbon sources, except when the adapted strain was tested back in glucose, showing thinnest fibers among all. The slight change in the fiber diameter could be due to changes in culture condition, which interfere with the formation of microfibers, resulting in smaller diameters, as observed by Li et al. (2021b). At cycle 30, a general increase in width of fiber dimension in all the conditions was detected, ranging from 34–54, 28–52, and 26–44 nm in 30MA, 30MG, and 30MF, respectively. Increasing in fiber dimension are correlated with an improvement of the mechanical properties. In BC produced by *Komagataeibacter* strains, an improvement in the G’and G” modules was observed (Numata et al., 2019).

The XRD diffraction was applied to assess crystallinity changes during the ALE. The X-ray diffractograms of the BC in all conditions showed the characteristic peaks at the angle of 14.5, 16.5, and 22.5 (Supplementary Figure S1) with slight differences in intensity. The least crystallinity index was observed in GC condition (79.35%), whereas MF and MG in the cycle 1 showed the highest crystallinity (95.69 and 91.75%, respectively). Among the adaptation cycles, no differences were observed in the crystallinity terms. Only when the adapted strain was cultivated in a different carbon source than mannitol (MG and MF conditions at cycle 30), a decrease in the crystallinity (84.81 and 89.20%, respectively) was observed. This result is in agreement with a previous study, in which similar decreasing pattern was observed when the *K*. *xylinus* MSKU12 adapted in coconut water was cultured in standard HS medium (Naloka et al., 2020).

## Conclusion

Different approaches have been applied to improve BC production. The strain selection and the utilization of the proper carbon source are the crucial points to set-up new processes for BC production. The greatest efforts to improve the BC yield are focused on testing alternative carbon sources, often exploiting waste materials, mainly to make the process more sustainable. In this study we adopted the ALE approach to improve the BC production by repeatedly cultivating *K. xylinus* K2G30 supplementing a different carbon source (D-mannitol) than glucose. We observed an improvement in BC of about 38% in the adapted strain compared to the parental one, followed by an increase in mannitol utilization for BC production.

These results show that the use of mannitol was considerable increased respect to glucose. This evidence is relevant considering the production of BC from waste containing mannitol. Moreover, the adaptation on mannitol did not strongly affect the ability of the strain to use glucose, since the BC yield resulted slightly decreased. Based on these considerations, the adaptation on mannitol did not occur at the expense of glucose, highlighting the versatility of K2G30 in producing BC from different carbon sources.

Furthermore, according to the metabolic pathway of mannitol, we assayed a second carbon source (fructose) and observed a substantial increase in the BC production. The improvements obtained for the tested carbon sources increase considerably the use of *K. xylinus* K2G30 for BC production, allowing the design of new complex media, including multiple carbon sources and/or waste as feedstock. The adaptation mechanism is a highly complex process, involving remodulation of the metabolic pathways, genetic mutations and translational events. Previous studies highlighted time-lapse of the adaptation events occurring during the early stage and in long-term evolutionary experiments. The *K. xylinus* K2G30 adaptation presented here is an ongoing experiment and will be used in the future to analyze short- and long-term adaptation events occurring in the shift of the carbon source. To this aim, the transcriptome analysis of the adapted strain will be performed. Furthermore, the genome sequencing of the adapted strain and the comparison with the parental one, can clarify if the observed phenotype changes are due to genetic alteration or to a transient phenotypic shift.

Integrating the ALE approach with the development of genetic engineering tools could provide the resources necessary to tune the adaptation mechanisms existing in bacteria with the need to obtain high cellulose producer strain.

## Data availability statement

The original contributions presented in the study are included in the article/Supplementary material, further inquiries can be directed to the corresponding author.

## Author contributions

KA: methodology, formal analysis, writing the original draft and data curation. SLC: conceptualization, investigation, validation, data visualization and analysis, supervision and writing—review and editing. MB: structural analysis and writing the original draft. SC: supervision and review and editing. MG: conceptualization, investigation, resources, review and editing, supervision, project administration, and funding acquisition. All authors contributed to the article and approved the submitted version.

## Funding

This research was supported by FAR Fondo di Ateneo per la Ricerca UNIMORE 2020 and EU Project “Implementation and Sustainability of Microbial Resource Research Infrastructure for the 21st Century” (IS_MIRRI21, Grant Agreement Number 871129).

## Conflict of interest

The authors declare that the research was conducted in the absence of any commercial or financial relationships that could be construed as a potential conflict of interest.

## Publisher’s note

All claims expressed in this article are solely those of the authors and do not necessarily represent those of their affiliated organizations, or those of the publisher, the editors and the reviewers. Any product that may be evaluated in this article, or claim that may be made by its manufacturer, is not guaranteed or endorsed by the publisher.
