# Supplementary material for: Better under stress: Improving bacterial cellulose production by Komagataeibacter xylinus K2G30 (UMCC 2756) using adaptive laboratory evolution
Source: Front Microbiol. 2022 Oct 12;13:994097. doi: 10.3389/fmicb.2022.994097 (PMC9605694; doi:10.3389/fmicb.2022.994097)
Supplement: Supplementary file 1 [file Data_Sheet_1.docx]

| **Cycles** | **MA** | | **MC** | | **GC** | | **MG** | |
| --- | --- | --- | --- | --- | --- | --- | --- | --- |
|  | GlcA | pH | GlcA | pH | GlcA | pH | GlcA | pH |
| Cycle 1 | 0.04 ± 0.02 | 6.25 ± 0.01 | 0.09 ± 0.01 | 6.32 ± 0.04 | 8.33 ± 0.65 | 3.6 ± 0.06 | 7.43 ± 1.79 | 3.55 ± 0.04 |
| Cycle 2 | 0.08 ± 0.07 | 6.16 ± 0.01 | 0.13 ± 0.02 | 6.17 ± 0.01 | 9.26 ± 1.23 | 3.55 ± 0.03 | 7.19 ± 1 | 3.5 ± 0.01 |
| Cycle 3 | 0.06 ± 0.06 | 6.06 ± 0.02 | 0.08 ± 0 | 6.03 ± 0.04 | 6.89 ± 1.91 | 3.49 ± 0.02 | 9 ± 1.05 | 3.45 ± 0.02 |
| Cycle 4 | 0.03 ± 0.04 | 6.31 ± 0.02 | 0.08 ± 0.06 | 6.29 ± 0.04 | 8.25 ± 2.53 | 3.69 ± 0.03 | 7.1 ± 1.14 | 3.63 ± 0.01 |
| Cycle 5 | -0.02 ± 0.09 | 6.34 ± 0.01 | 0.09 ± 0.02 | 6.22 ± 0.03 | 7.58 ± 1.23 | 3.67 ± 0.04 | 8.67 ± 0.66 | 3.61 ± 0.02 |
| Cycle 6 | -0.06 ± 0.04 | 6.38 ± 0.02 | 0.06 ± 0.02 | 6.26 ± 0.01 | 7.35 ± 2.08 | 3.69 ± 0.03 | 9.92 ± 2.61 | 3.63 ± 0.02 |
| Cycle 7 | 0.06 ± 0.05 | 6.43 ± 0.04 | 0.04 ± 0.05 | 6.29 ± 0.01 | 7.75 ± 1.74 | 3.72 ± 0.02 | 7.47 ± 1.67 | 3.65 ± 0.03 |
| Cycle 8 | 0.06 ± 0.03 | 6.42 ± 0.03 | -0.01 ± 0.07 | 6.29 ± 0.01 | 5.57 ± 2.09 | 3.72 ± 0.02 | 7.29 ± 1.51 | 3.65 ± 0.02 |
| Cycle 25 | 0.09 ± 0.04 | 6.41 ± 0.02 | 0.01 ± 0.02 | 6.19 ± 0.02 | 10.07 ± 2.14 | 3.73 ± 0.03 | 8.54 ± 1.44 | 3.65 ± 0.04 |
| Cycle 28 | -0.04 ± 0.01 | 6.41 ± 0.03 | -0.05 ± 0.01 | 6.26 ± 0.05 | 6.11 ± 0.22 | 3.66 ± 0.12 | 6.6 ± 1.23 | 3.59 ± 0.13 |
| Cycle 30 | 0.08 ± 0.02 | 6.37 ± 0.05 | 0.01 ± 0.05 | 6.27 ± 0.02 | 7.65 ± 0.77 | 3.49 ± 0.02 | 4.87 ± 0.45 | 3.39 ± 0.01 |

**Tables**

**Supplementary Table 1** Production of gluconic acid among cycle of adaptation and change in pH of culture medium after five days of incubation; Data were reported as mean ± standard deviation

**Supplementary Table 2** Comparison between wild type and 30 cycle adapted strain in fructose in terms of BC yield, consumed carbon source, gluconic acid and pH. All the data were represented as mean ± standard deviation

| **Condition** | **BC yield (g/L)** | **Consumed CS (g/L)** | **Gluconic acid (g/L)** | **pH** |
| --- | --- | --- | --- | --- |
| 1MF | 2.24 ± 0.32 | 6.76 ± 0.21 | 0.13 ± 0.03 | 5.9 ± 0.02 |
| 30MF | 3.47 ± 0.11 | 10.6 ± 1.04 | 0.68 ± 0.48 | 6.18 ± 0 |

**Figures**

**
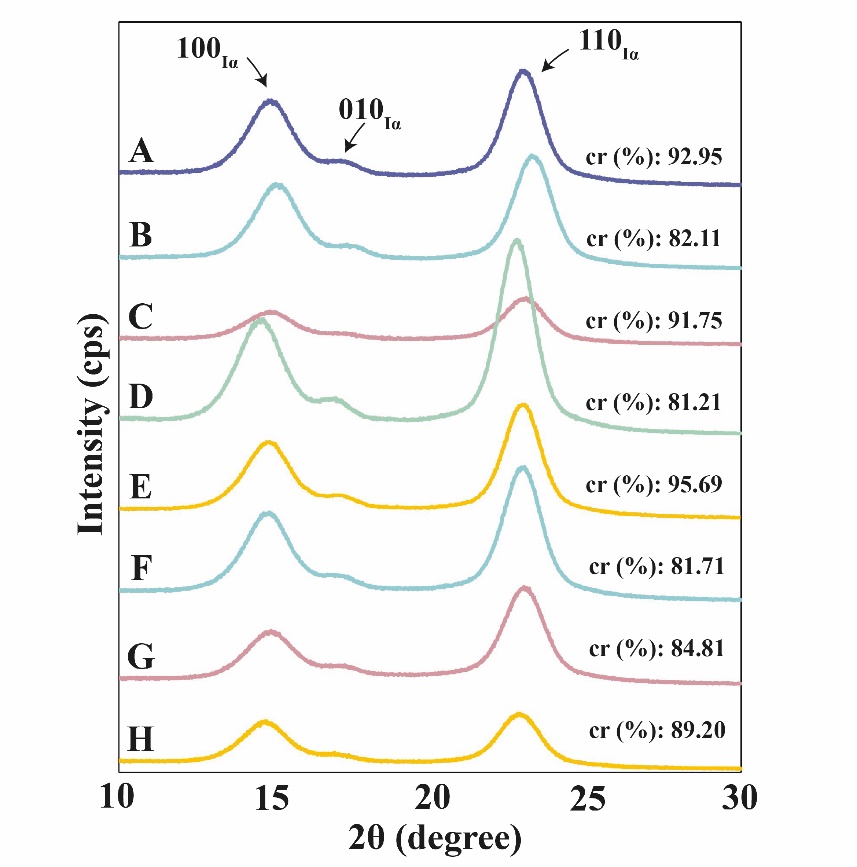
**

**Figure S1** BC X-ray diffraction spectra of (A) 1GC, (B) 1MA, (C) 1MG, (D) 1MC, (E) 1MF, (F) 30MA, (G) 30MG, (H) 30MF. The spectra for each conditions show the characteristic peaks of BC at 14.5º, 16.6º and 22.7º of 2θ.
